# Brief case management versus usual care for frequent users of emergency departments: the Coordinated Access to Care from Hospital Emergency Departments (CATCH-ED) randomized controlled trial

**DOI:** 10.1186/s12913-016-1666-1

**Published:** 2016-08-24

**Authors:** Vicky Stergiopoulos, Agnes Gozdzik, Jason Tan de Bibiana, Tim Guimond, Stephen W. Hwang, Donald A. Wasylenki, Molyn Leszcz

**Affiliations:** 1 Centre for Urban Health Solutions, St. Michael’s Hospital, Toronto, Canada; 2 Department of Psychiatry, University of Toronto, Toronto, Canada; 3 Centre for Addiction and Mental Health, Toronto, Canada; 4 Division of General Internal Medicine, Department of Medicine, University of Toronto, Toronto, Canada; 5 Mount Sinai Hospital, Toronto, Canada

**Keywords:** Randomized controlled trial, Case management, Emergency department, Frequent users, Mental health, Addictions

## Abstract

**Background:**

Frequent users of hospital emergency departments (EDs) are a medically and socially vulnerable population. This article describes the rationale for a brief case management intervention for frequent ED users with mental health and/or addiction challenges and the design of a randomized trial assessing its effectiveness.

**Methods/Design:**

Eligible participants are adults in a large urban centre with five or more ED visits in the past year, with at least one prior visit for a mental health or addictions reason. Participants (*N* = 166) will be randomized to either 4 to 6 months of brief case management or usual care, and interviewed every 3 months for 1 year. Consent will be sought to access administrative health records. A subset of participants (*N* = 20) and service providers (*N* = 13) will participate in qualitative data collection.

**Discussion:**

Addressing the needs of frequent ED users is a priority in many jurisdictions. This study will provide evidence on the effectiveness of brief case management, compared to usual care, on reducing ED visits among frequent ED users experiencing mental health or substance misuse problems, and inform policy and practice in this important area.

**Trial registration:**

ClinicalTrials.gov Identifier: NCT01622244. Registered 4 June 2012.

## Background

In most jurisdictions, a small subgroup of the population is responsible for a disproportionately large share of hospital emergency department (ED) visits. A systematic review of studies from the United States found that patients who visited the ED four or more times per year accounted for 4.5 % to 8 % of all ED patients and 21 % to 28 % of all ED visits [1]. Although the threshold for frequent ED use varies amongst studies, many authors select a cut-off of more than four visits per year [2], which according to Locker et al. [3] correspond to a non-random event, and could be justified as a common threshold to improve comparison between studies in this area.

Contrary to widely-held views, frequent ED users represent a heterogeneous group of service users [1]. Some individual-level factors associated with increased risk of frequent ED use in the U.S. include being a woman, being black and having Medicare or Medicaid; nonetheless, White people with insurance continue to comprise the largest absolute number of frequent ED users in the US [1]. Frequent ED peaks at three points in the lifespan: among children, those aged 25–44 years and those aged 65 or more [1]. Some frequent users repeatedly visit the same ED, while others present to several different EDs [1, 4, 5]. Although seemingly contradictory, research consistently shows that frequent ED users are also frequent users of other health services, and access to primary care in particular appears to be high [1]. However, lack of satisfaction with their physician, inability to receive timely access to care in the community, complexity of needs, and a personal preference for the ED over a physician’s office may be key drivers of why frequent ED users preferential choose the ED as a source of care [1]. It is also important to note that not all ED visits are preventable or treatable in a non-acute setting, particularly those addressing critical concerns.

The most common complaints reported by frequent ED users are likewise varied. Compared to occasional ED users, however, frequent ED users experience poorer physical health [1] and face many health and social challenges, including mental illness and/or addiction, acute and chronic medical conditions, social isolation, limited socio-economic resources, and homelessness [4, 6–9].

Thus far, the most commonly studied intervention for frequent ED users has been case management [2, 10], generally entailing the coordination of appropriate services and providers for an individual by a designated point of contact. Case management can include a collaborative process of assessment, individualized care planning, navigation, and advocacy [2, 11, 12]. Two prior systematic reviews have examined the effectiveness of interventions for frequent ED users among the “general” population (studies focusing specifically on subpopulations or on specialized care settings were excluded) [2, 10]. Althaus et al. [2] reported that case management reduced ED cost and improved social and clinical outcomes, while Soril et al. [10] noted significant reductions in ED use in 9 out of 12 studies of case management, including two randomized controlled trials [13, 14]. A recently published third systematic review of ED visit reduction programs in the U.S. noted that there was sufficient evidence from moderate to high quality studies to conclude that case management was the only intervention that consistently reduced ED use [15].

Despite the mounting evidence on the effectiveness of case management for frequent ED users, there remain important gaps in our knowledge base. First, for most individuals who meet the “frequent user” threshold in a given year, the experience is temporary or episodic and their ED use may decrease in the following year without intervention [4, 5, 7, 16]. This is an important threat to validity in non-experimental studies of interventions for frequent ED users. Second, the case management interventions studied thus far are heterogeneous with respect to the types of case management strategies and services offered, staff composition (e.g. independent case managers, multidisciplinary team, and/or with physicians), duration of follow-up [2, 10, 11] and unique program-specific elements (e.g. telephone-based case management [14] and case management with drop-in group appointments [17]). Third, there is limited evidence on the effectiveness of case management for frequent ED users who present with mental health and/or addictions needs [12]. Previous studies have excluded participants with psychotic disorder [14] or severe substance misuse [18, 19], and a recent systematic review and meta-analysis evaluating care coordination strategies (including case management) for frequent users of health care services found that these strategies were only effective in reducing hospital admissions for patients with chronic conditions other than mental illness [12]. Thus, frequent ED users with mental health and substance related conditions may have more complex needs and require comprehensive and assertive case management supports [11]. Prior research has shown that time limited case management strategies can significantly reduce hospitalization [20] and homelessness [21] among people with mental illness, however, little is known if such programs can successfully reduce ED visits among frequent users of EDs who present with mental health and/or substance misuse problems.

In order to address existing knowledge gaps and generate evidence to inform policy and practice, the Coordinated Access to Care from Hospital Emergency Departments (CATCH-ED) trial was initiated to implement and evaluate a brief case management intervention for frequent ED users with mental health and/or addiction challenges in Toronto, Canada’s largest urban centre. The primary study objective is to assess the effectiveness of a brief intervention in reducing ED utilization for the target population. A secondary objective is to assess the effectiveness of the intervention in improving health outcomes. We hypothesized that the intervention will be associated with reductions in the rate of ED visits, reduced symptom and substance use severity, increased health-related quality of life, and a reduced number of days in hospital compared to usual care, after 12 months.

The purpose of this article is to briefly describe the CATCH-ED intervention and study protocol and to describe the strengths, limitations and potential implications of the trial. Subsequent articles will report on the primary and secondary study outcomes and the perspectives and experiences of service users and service providers with the intervention.

## Methods/Design

### Trial design

**Fig. 1:**
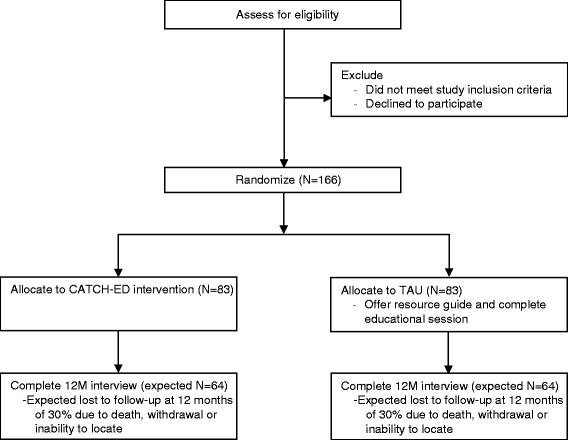
Expected study flow diagram

CATCH-ED is a non-blinded, parallel-group, randomized controlled trial of a brief case management intervention implemented across 6 hospital EDs in Toronto, Canada. Participants will be randomly assigned to the CATCH-ED intervention group or treatment as usual (TAU) control group, and followed for 12 months (Fig. 1).

Quantitative data on health service use and health outcomes will be collected for all study participants during 12 months of follow-up. Qualitative data on the experience of service providers and of a subset of participants in the intervention group will also be collected through interviews and a focus group.

### Intervention and usual care

In the intervention group, participants will receive brief case management over four to six months. TAU participants will receive care as usual in the community, as well as an educational session and resource guide outlining available community-based services.

The CATCH-ED intervention was informed by the Critical Time Intervention (CTI) model, an empirically supported, time-limited case management intervention designed to prevent homelessness and other adverse outcomes for people with mental illness following discharge from hospital, prison, shelters, and other institutions [18–26]. Similar to the CTI model, participants in the CATCH-ED intervention group will receive case management support in three phases over four to six months of follow-up, focused first on engagement and goal-setting, followed by bridging and connecting to community-based services, and finally transitioning and transferring care to longer-term services as needed (Table 1). Seconded from three community mental health agencies, CATCH-ED case managers will have access to a range of community support options, including primary care, peer support, mental health and addictions counselling, and other health and social services as needed, through partnerships with four community health centres and one peer outreach agency. The case managers will be overseen by a program manager and offer outreach, home visits, crisis intervention, supportive therapy, practical needs assistance and care coordination, aiming to integrate hospital, community and social care and improve continuity of care. CATCH-ED case managers will maintain small caseloads of approximately 15 clients each.

**Table 1:** Description of the phases of the CATCH-ED intervention

Phases	Description
Phase 1 (Month 1–2): Engagement and goal-setting	- Engage participant as soon as it is feasible (meet in the hospital ED or inpatient unit if possible) - Rapidly assess needs, strengths, and existing resources - Understand reasons for frequent ED use - Provide practical needs assistance to meet basic needs and resolve immediate crises - Develop individualized and focused treatment and support plans - Liaise with participant’s other care providers
Phase 2 (Months 2–3): Bridging and connecting to community-based care	- Provide ongoing practical needs assistance and crisis support - Make referrals to longer-term community-based supports as needed - Strengthen support network as needed
Phase 3 (Months 3–6): Transitioning and transfer of care	- Transfer care to longer-term resources and support network developed in Phase 2

### Participants

Eligible participants are adults with five or more ED visits in the past year, to any one of the 6 participating hospital EDs, with at least one of those visits for a mental health or addictions-related concern. ED clinicians at participating hospitals will identify frequent ED users through either frequent user lists or automated flagging systems, and refer them to the study team. A member of the research team will assess participant study eligibility, and obtain informed consent before study enrolment.

### Randomization

Randomization will be performed via computer by the study data coordination centre at the completion of the eligibility screening and baseline interview, and the decision will be directed to the research staff’s tablet computer and communicated to participants. Participants randomized to the intervention group will be immediately referred to a CATCH-ED case manager. The randomization sequence will assign participants to either the intervention or the TAU group using block randomization, with a 1:1 allocation ratio and randomly selected block sizes. This technique will maintain balanced group sizes between the intervention and the TAU group at intermediate points in the recruitment process and minimize the possibility of study staff predicting group assignment [27].

### Blinding

Due to the nature of the intervention, participants, service providers, and research staff involved with collecting and assessing outcome data will not be blinded (i.e. they were aware of group assignment to either the intervention or the TAU group) for the duration of the study.

### Data collection and retention strategies

During the 12-month follow-up period, participants will meet with a member of the research team to complete a series of questionnaires at 3-month intervals. The questionnaires include measures on participants’ self-reported health service use and health status. Interviews will be conducted in various locations, based on participant preference, including the research team’s office, service provider locations, and public settings. Responses will be uploaded immediately to the data coordinating centre’s secure server using tablet computers.

The duration for the baseline, 6-month, and 12-month interviews will be approximately one hour, while the 3-month and 9-month interviews will be approximately 15 min. Participants will receive a cash honorarium and public transit tokens for completing each follow-up interview. To reduce loss to follow-up, the research team will collect and update contact information for participants and their family, friends, and service providers at each follow-up interview and during monthly check-ins in between scheduled interviews.

Participants will also be asked to provide consent to use their provincial health card number to access administrative health databases for the 12-month period before and after study enrolment at the Institute for Clinical and Evaluative Sciences (ICES), where health service use data for all residents in the province of Ontario is stored.

### Measures

Table 2 displays the full list of study measures and timelines for data collection. The primary outcome measure is the rate of ED visits during the 12-month period following study enrolment. Secondary outcomes include mental health symptom severity (CSI), addiction severity (ASI), and health-related quality of life (SF-12 and EQ-5D) at 12 months after study enrolment, and the rate of days in hospital during the 12-month period following study enrolment. Exploratory outcomes include disease-specific quality of life (QoLI-20) at 12 months after study enrolment, and the rate of hospital admissions during the 12-month period following study enrolment.

**Table 2:** Study measures

Study measure	Data collection timeline	References
*Health service use outcomes*		
Health Service Use Inventory	BL, 3M, 6M, 9M, 12M	[28–35]
Administrative Health Data	12-months before BL to 12-months after BL	
*Health outcomes*		
Short Form Health Survey, 12-item (SF-12)	BL, 6M, 12M	[36–38]
Euro-Qol 5D (EQ-5D)	BL, 6M, 12M	[38–41]
Quality of Life Index, 20-item (QoLI-20)	BL, 6M, 12M	[42, 43, 63]
Colorado Symptom Index, modified (CSI)	BL, 6M, 12M	[44–47]
Addiction Severity Index (ASI)	BL, 6M, 12M	[48]
*Additional measures*		
Socio-demographic questions	BL	
Comorbid Conditions (CMC) list	BL, 3M, 6M, 9M, 12M	[47, 49, 50]
Diagnostic information	BL, 6M, 12M	
Access to Care questions	BL, 6M, 12M	[50, 51, 64]
Working Alliance Inventory-Participant (WAI-PAR)	3M, 6M	[52–56]

*Abbreviations BL* Baseline interview, *3M* 3-month interview, *6M* 6-month interview, *9M* 9-month interview, *12M* 12-month interview

#### Health service use outcomes

Participants will be asked questions about their health service use, including the number of ED visits, days in hospital, and hospital admissions in the past 6 months, using an inventory adapted from the At Home/Chez Soi trial [28–35].

Self-reported service use data will be validated by linkage to administrative health service use databases at ICES for the 12-month period before and after study enrolment for consenting participants.

#### Health outcomes

Short Form Health Survey, 12-item (SF-12): The SF-12 is a brief, self-report measure of generic health status. The 12 items of the SF-12 produce a physical component summary score and a mental component summary score which range between 0 and 100 [36–38].

EuroQol-5D (EQ-5D): The EQ-5D is a generic measure of health-related quality of life and the most frequently used instrument in the calculation of quality adjusted life years (QALY) for cost-utility analyses [38–41]. The EQ-5D includes five items concerning mobility, self-care, usual activities, pain/discomfort, and anxiety/depression that are weighted to produce a single utility score between 0 and 1. The Visual Analog Scale (VAS) of the EQ-5D will also be included, which will allow participants to rate their overall health, mental health and physical health from 0 to 100.

Quality of Life Index, 20-item (QoLI-20): The QoLI-20 is a structured self-report interview designed to assess quality of life for people with severe mental illness. There are 7 subjective scales (living situation, everyday activities, family, social relationships, finances, safety, and satisfaction with life in general) and 4 objective scales (everyday activities, enough money, family contacts, and contacts with friends). The 20-item version was developed by Uttaro and colleagues and yields a score ranging from 20 to 140 [42, 43].

Colorado Symptom Index (CSI), modified: The CSI is a 14-item instrument designed specifically for individuals with mental health problems to assess the presence and frequency of symptoms of mental illness experienced within the past month [44–47]. The 14 items of the CSI yield a summary score that ranges between 0 and 56.

Addiction Severity Index (ASI): The ASI is a semi-structured interview for research or clinical assessment that is designed to assess seven potential problem areas with substance-abusing patients: medical status, employment and support, drug use, alcohol use, legal status, family/social status, and psychiatric status [48]. The drug use and the alcohol use module will be administered to yield composite scores measuring the severity of recent drug and alcohol use, ranging from 0 to 1.

For all above instruments, with the exception of the ASI and CSI, higher scores are indicative of better outcomes. For the ASI and CSI, higher scores indicate poorer outcomes (e.g. greater symptom severity).

#### Additional measures

Socio-demographic characteristics: Participants will be surveyed about their socio-demographic characteristics, housing, employment, and income support status.

Comorbid Conditions (CMC) list: Participants will be asked to complete a checklist of chronic medical conditions, developed from the Canadian Community Health Survey and the National Population Health Survey [47, 49, 50].

Diagnostic information: Participants will be asked if they had ever been diagnosed by a medical professional, for a list of psychiatric disorders.

Access to Care: Participants will be asked to complete questions about their access to a regular source of medical care, based on questions from the Canadian Community Health Survey and the Street Health Survey [50, 51].

Working Alliance Inventory-Participant (WAI-PAR): Participants will be asked to complete the 12-item WAI-PAR questionnaire, to assess how they think and feel about the therapeutic relationship with their case manager [52–56]. The WAI-PAR includes three subscales relating to task, goal and bond, and yields a summary score that ranges from 12 to 84, with higher scores indicating a stronger therapeutic relationship.

Program utilization data will be captured from program records, including the number of contacts with case managers and length of time in the program.

### Sample size

The primary outcome is the rate of ED visits during the 12-month period following study enrolment, with a rate reduction of 20 % considered the minimum difference of clinical significance.

The sample size calculation for this study considered three important factors: (1) variable rates of ED use may contribute to observed changes over time, since eligible participants are at the extreme of the distribution of ED visits for the overall population, (2) accessing emergency services may provide treatment that contributes to observed changes over time, and (3) the distribution of ED visits tends to be highly right skewed, following a negative binomial or another over dispersed count distribution. To account for these factors, the distribution and natural reduction of ED visits over time for frequent users were estimated using data from the control group of an earlier randomized trial [13] among frequent ED users with 5 or more visits in the past year, and the sample size calculation was performed using the formula derived by Tango [57] for Poisson-type outcomes and verified by a simulation study.

Ultimately, this sample size calculation proposed that a sample size of 64 participants in each arm would allow for the detection of a 20 % rate reduction. Allowing for up to 30 % attrition in participant follow-up, the final sample size was set at 83 participants in each arm, for a total of 166 participants.

### Statistical methods

Descriptive statistics will be used to describe study participants’ socio-demographic and clinical characteristics.

The analyses of primary, secondary, and exploratory outcomes will adopt an intention-to-treat approach, to compare 12-month outcomes between participants randomized to the intervention and TAU groups.

For outcomes expressed as count variables (number of ED visits, number of days in hospital, number of hospital admissions), Poisson or negative binomial regression (depending on the variance of the outcome distribution) will be used to model the rate ratio for the intervention group compared to the TAU group during the 12-month period following study enrolment, with two-sided *p* values and 95 % confidence intervals. The rates from the self-reported data will be modelled for the 6-month period from 6 to 12 months following study enrolment (from the 12-month interview) and for the 12-month period from 0 to 12 months following study enrolment (by combining the 6-month and 12-month interview). Each regression model will include as a covariate the corresponding baseline rate value, which captures the period prior to study enrolment. Administrative data will be used to validate the self-reported data for all health service use outcomes.

For outcomes expressed as continuous variables (SF-12 physical and mental component summary scores, EQ-5D utility score, CSI summary score, ASI drug and alcohol scores), linear regression will be used to model the average difference for the intervention group compared to the TAU group at 12 months following study enrolment, with two-sided p values and 95 % confidence intervals. The data for each outcome will be assessed to ensure a Gaussian distribution, and each regression model will include the corresponding baseline value as a covariate.

### Qualitative methods

#### Participant recruitment and data collection

The qualitative study component will recruit 20 intervention group participants and 13 CATCH-ED service providers to participate in an interview or a focus group to explore their experience with the intervention. Intervention group participants will be recruited at their 6-month interview and sampled purposively to include participants referred from different EDs. CATCH-ED case managers and clinicians (family physicians and mental health counsellors) and managers from the partner organizations will also be recruited to the qualitative study component.

Qualitative data collection, using a semi-structured interview guide with open-ended questions and probes, will be conducted by one member of the research team. Intervention group participants will receive an additional honorarium and public transit tokens for their participation.

#### Qualitative analysis

The primary approach for qualitative data analysis will be thematic analysis of audio-recorded transcripts from the interviews and focus group [58, 59] by three members of the research team. The research team will identify key concepts and code transcripts through an iterative process of classifying, comparing, grouping, and refining the key concepts into themes [58, 60, 61], using QSR NVivo 10 software to manage and analyze the data.

## Discussion

The CATCH-ED trial aims to assess the effectiveness of a promising brief intervention for frequent ED users with mental health and/or addiction challenges, a population over-represented among frequent ED users in many jurisdictions. Given the limited evidence on the effectiveness of case management for frequent ED users, in particular the small number of randomized controlled trials and the disparate findings for frequent users with mental health and/or addictions needs [2, 11, 12] the CATCH-ED trial will yield important new information to inform policy and practice for this vulnerable population.

The study has several strengths, including a well described intervention and rigorous methods for assessing its effectiveness. CATCH-ED was informed by the CTI model, a well-defined case management model that can be implemented in various service delivery contexts [18–26]. The study uses a randomized controlled design and validated measures to assess health outcomes, will obtain in-depth qualitative data on the perspectives and experiences of both participants and service providers with the intervention, and will access province-wide administrative health service utilization data repositories to assess participant acute care utilization. Our statistical analyses will account only for baseline differences in the outcomes of interest between intervention and TAU groups and will assume that randomization has balanced the distribution of all other confounders equally among the intervention and TAU groups. The use of automatic ED flagging systems and support of ED staff in facilitating timely identification of frequent ED users at referring hospital EDs will improve study recruitment and help ensure that only eligible participants are referred to the study.

Building on lessons learned from previous longitudinal research with disadvantaged and hard-to-reach groups [62], we hope to retain consistently high follow-up rates. We’ll employ a variety of strategies, including completing regular check-ins with participants to update contact information and schedule upcoming interviews, maintain relationships with a network of community health and social service providers serving study participants, and conduct outreach for hard-to find participants.

CATCH-ED will be implemented in 6 EDs in a single urban centre within a system of universal access to health care, which may limit the generalizability of our findings to other settings. In alignment with previously published studies, we use study inclusion criteria of five or more ED visits in the previous year, consistent with the cut-off proposed by Locker et al. [3]. However, as participants will be identified and referred based on ED visits in a single ED, some may have had much higher rates of ED use if they accessed more than one ED, not an uncommon finding in this population [1, 4, 5].

Addressing the needs of frequent ED users and reducing avoidable acute care utilization is a priority in many jurisdictions. The CATCH-ED trial will evaluate the effectiveness of a brief case management intervention for frequent ED users with mental health and addiction challenges, yielding important new information to better inform policy and practice for this vulnerable population.

## Abbreviations

ASI: Addiction Severity Index
CATCH-ED: Coordinated Access to Care from Hospital Emergency Departments
CMC: Co-morbid conditions
CSI: Colorado Symptom Index
CTI: Critical Time Intervention
ED: Emergency department
EQ-5D: EuroQol-5D
ICES: Institute for Clinical and Evaluative Sciences
QALY: Quality adjusted life years
SF-12: Short Form Health Survey, 12-item
TAU: Treatment as usual
WAI-PAR: Working Alliance Inventory-Participant
